# Frequency of subclinical interstitial lung disease in COVID-19 autopsy cases: potential risk factors of severe pneumonia

**DOI:** 10.1186/s12890-023-02692-1

**Published:** 2023-10-27

**Authors:** Hiromichi Iwashita, Yoshinori Kawabata, Hiroyuki Hayashi, Shoichiro Matsushita, Tsuneo Yamashiro, Mai Matsumura, Yukihiro Yoshimura, Toshiaki Kataoka, Hideaki Mitsui, Takehisa Suzuki, Toshihiro Misumi, Tomonori Tanaka, Sosuke Ishijima, Junya Fukuoka, Tae Iwasawa, Takashi Ogura, Koji Okudela

**Affiliations:** 1 Department of Pathology, School of Medicine, Yokohama City University, 3-9 Fukuura, Kanazawa-ku, Yokohama-shi, Kanagawa 236-0004 Japan; 2 Department of Pathology, Saitama Cardiovascular and Respiratory Center, 1696, Itai, Kumagaya-shi, Saitama, 360-0197 Japan; 3 Division of Pathology, Yokohama Municipal Citizen’s Hospital, 1-1 Mitsuzawanishimachi, Kanagawa-ku, Yokohama-shi, Kanagawa 221-0855 Japan; 4 Department of Radiology, Yokohama City University Hospital, 3-9 Fukuura, Kanazawa-ku, Yokohama-shi, Kanagawa 236-0004 Japan; 5 Division of Infectious disease, Yokohama Municipal Citizen’s Hospital, 1-1 Mitsuzawanishimachi, Kanagawa-ku, Yokohama-shi, Kanagawa 221-0855 Japan; 6 Department of Biostatistics, Yokohama City University School of Medicine, 3-9 Fukuura, Kanazawa-ku, Yokohama-shi, Kanagawa 236-0004 Japan; 7 Department of Diagnostic Pathology, Kobe University Hospital, 7-5-2 Kusunoki-cho, Chuo-ku, Kobe-shi, Hyogo 650-0017 Japan; 8 Department of Pathology Informatics, Nagasaki University Graduate School of Biomedical Sciences, 1-14 Bunkyo-machi, Nagasaki-shi, Nagasaki, 852-8521 Japan; 9 Division of Radiology, Kanagawa Cardiovascular and Respiratory Center, 6-16-1 Tomioka- higashi, Kanazawa-ku, Yokohama-shi, Kanagawa 236-0051 Japan; 10 Division of Respiratory Medicine, Kanagawa Cardiovascular and Respiratory Center, 6-16-1 Tomioka-higashi, Kanazawa-ku, Yokohama-shi, Kanagawa 236-0051 Japan

**Keywords:** Acute exacerbation, Coronavirus disease 2019 (COVID-19), Interstitial lung abnormalities, Interstitial lung disease, Usual interstitial pneumonia

## Abstract

Risk factors of severe coronavirus disease 2019 (COVID-19) have been previously reported; however, histological risk factors have not been defined thus far. The aim of this study was to clarify subclinical hidden interstitial lung disease (ILD) as a risk factor of severe pneumonia associated with COVID-19. We carefully examined autopsied lungs and chest computed tomography scanning (CT) images from patients with COVID-19 for interstitial lesions and then analyzed their relationship with disease severity. Among the autopsy series, subclinical ILD was found in 13/27 cases (48%) in the COVID-19 group, and in contrast, 8/65 (12%) in the control autopsy group (*p* = 0.0006; Fisher’s exact test). We reviewed CT images from the COVID-19 autopsy cases and verified that subclinical ILD was histologically detectable in the CT images. Then, we retrospectively examined CT images from another series of COVID-19 cases in the Yokohama, Japan area between February–August 2020 for interstitial lesions and analyzed the relationship to the severity of COVID-19 pneumonia. Interstitial lesion was more frequently found in the group with the moderate II/severe disease than in the moderate I/mild disease (severity was evaluated according to the COVID-19 severity classification system of the Ministry of Health, Labor, and Welfare [Japan]) (moderate II/severe, 11/15, 73.3% versus moderate I/mild, 108/245, 44.1%; Fisher exact test, *p* = 0.0333). In conclusion, it was suggested that subclinical ILD could be an important risk factor for severe COVID-19 pneumonia. A benefit of these findings could be the development of a risk assessment system using high resolution CT images for fatal COVID-19 pneumonia.

**Supplementary Information:**

The online version contains supplementary material available at 10.1186/s12890-023-02692-1.

## Introduction

The novel coronavirus infection (coronavirus disease 2019; COVID-19) has been spreading worldwide since the end of 2019. As of November 2022, the number of infected patients and deaths had reached approximately 600 million and 6.6 million, respectively. In Japan, 23 million people have been infected with the virus, and approximately 47,000 people have died.

Risk factors for severe COVID-19, such as male sex, old age, smoking history, obesity, and certain underlying diseases (hypertension, diabetes, and chronic respiratory disease), have been reported [1–3]. Among chronic respiratory diseases, chronic obstructive pulmonary disease [1–3] and interstitial lung disease (ILD) have been implicated [4–8].

A widely recognized path1ological feature of COVID-19 pneumonia is diffuse alveolar damage (DAD). Interestingly, DAD is also observed in acute exacerbations of interstitial pneumonia, with many cases linked to interstitial pulmonary fibrosis (IPF) or usual interstitial pneumonia (UIP). Notably, these two diseases share a crucial similarity in their histological findings [9–17]. Moreover, viral infections are a common trigger for DAD [17–20]. Therefore, we hypothesize that some cases of severe COVID-19 could represent acute exacerbations of subclinical ILDs triggered by severe acute respiratory syndrome coronavirus 2.

To verify this hypothesis, we carefully examined autopsied lungs and computed tomography (CT) images from COVID-19 cases and controls for hidden ILD lesions and analyzed their relationship with disease severity.

## Materials and methods

### Patients

#### Histopathological study

**Fig. 1:**
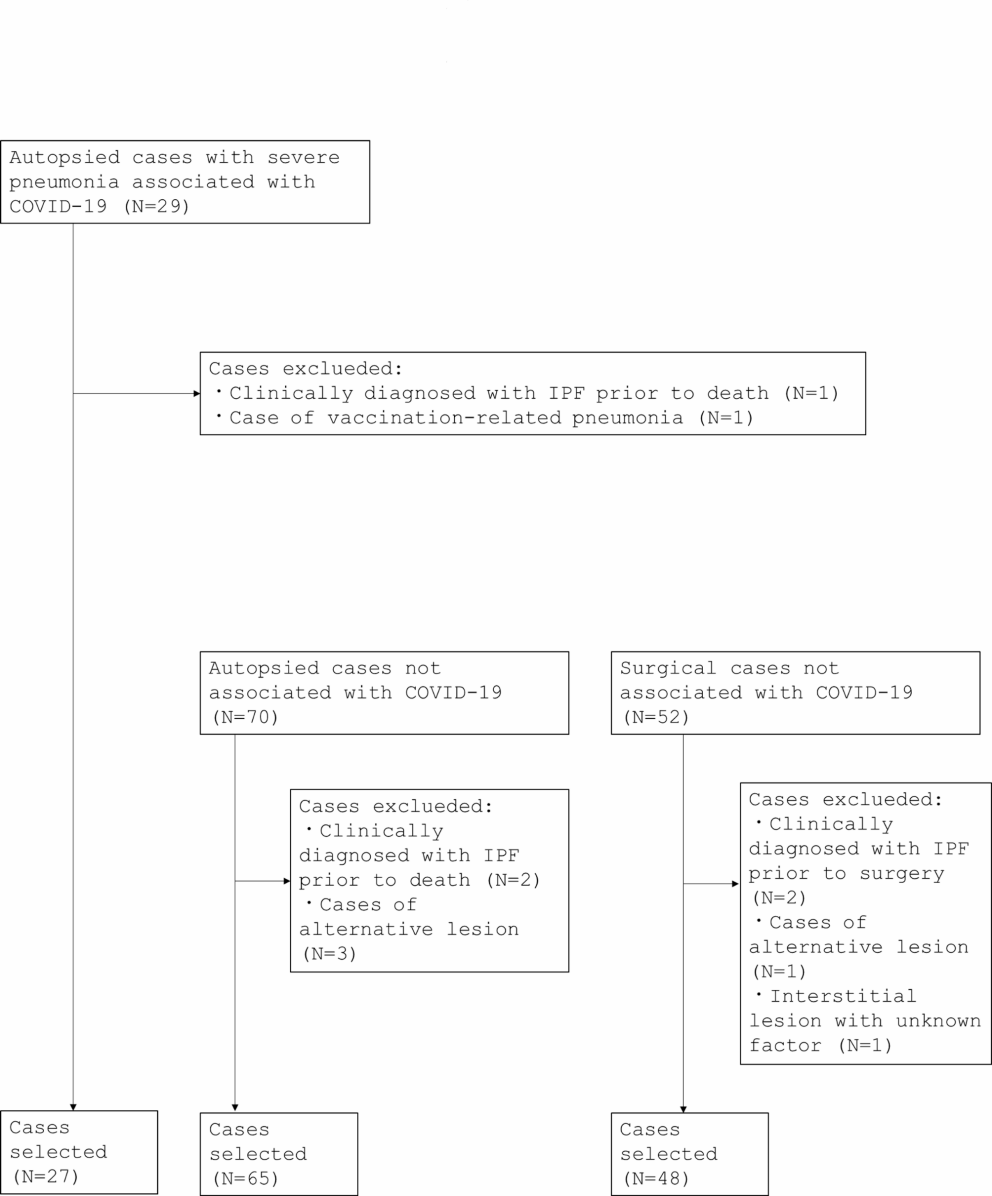
A flowchart for the subjected cases selection. We collected 29 COVID-19 autopsy cases, 70 control autopsy cases, and 53 control surgical cases. The selection process for the included cases is shown as a flow chart. An alternative lesion is an interstitial lesion with a histological pattern that is suggestive of other diseases (e.g., non-specific interstitial pneumonia and hypersensitive pneumonitis). COVID-19, coronavirus disease 2019; IPF, idiopathic pulmonary fibrosis

Twenty-seven COVID-19 autopsy cases (March 2020 to August 2021) were included in this study. For the control group, 65 non-COVID-19 autopsy cases (January 2017 to December 2021) and 48 post-surgical lower lobectomy cases (January 2017 to January 2021) were included. Lower lobectomy was performed to treat cancer in all 48 patients. All patients did not have clinically diagnosed ILD. A flow chart of the case selection is presented in Fig. 1. The patients’ characteristics at baseline are summarized in Table 1 and S1 Table.

**Table 1:** Basic patient characteristics in COVID-19 autopsy cases and control cases

	COVID-19 autopsy cases (N = 27)	Non-COVID-19 autopsy cases (N = 65)	Non-COVID-19 surgical cases (N = 48)
**Parameters**	**% (N)**	**% (N)**	**% (N)**
**Age, years**			
**mean ± SD**	79 ± 11	76 ± 7	73 ± 7
**median (range)**	81 (54–93)	77 (51–90)	74 (52–83)
**Sex**			
**Male**	62.96 (17)	60.00 (39)	66.67 (32)
**Female**	37.04 (10)	40.00 (26)	33.33 (16)
**M/F ratio**	1.7	1.5	2.0
**Smoking history**			
**Yes**	55.56 (15)	47.69 (31)	70.83 (34)
**No**	11.11 (3)	33.85 (22)	29.17 (14)
**Unknown**	33.33 (9)	18.46 (12)	0.00 (0)

Basic patient characteristics (such as age, sex, and smoking history) in COVID-19 autopsy and control cases are shown.

#### Radiological study

Chest CT images from 260 patients with COVID-19 (February to August 2020) were included.

### Histopathological examination

**Fig. 2:**
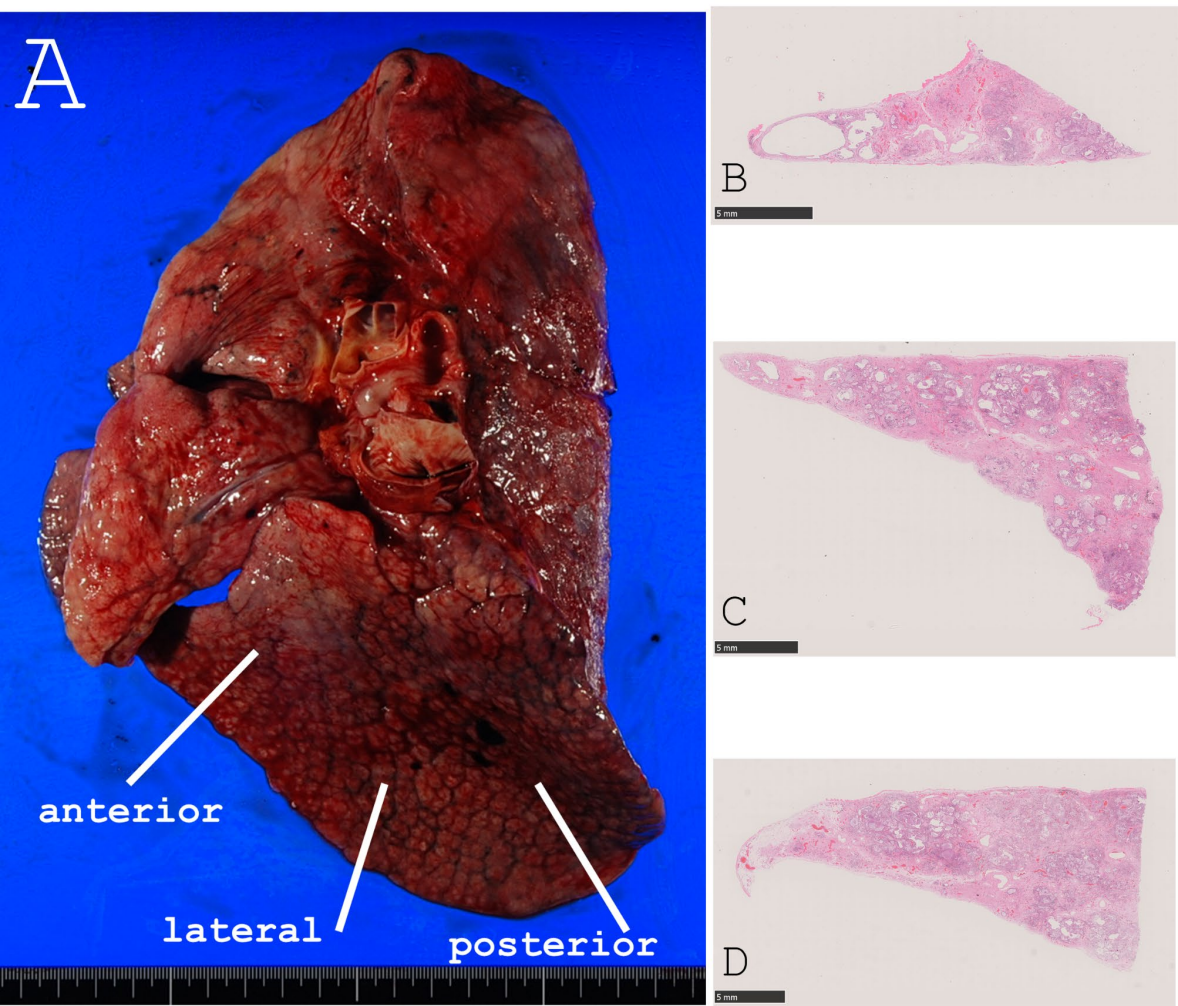
Specimen preparation for the histological examination. Gross appearance of the lung in a COVID-19 autopsy case (Left: pre-fixation, right: histological image). Vertical sections were obtained from three sites (anterior, lateral, and posterior) in the base of the lower lobe of the lung **(A)**. Histological images of the three sections are shown (**B:** anterior, **C:** lateral, **D:** posterior)

Tissue specimens were collected from the lower lobes of the lungs, where three specimens were vertically cut from the anterior, lateral, and posterior positions, as shown in Fig. 2. The specimens were collected from both sides in the autopsy cases and from one side in the surgical cases. Formalin-fixed paraffin-embedded tissue sections were stained with hematoxylin and eosin and with special staining methods such as Elastica van Gieson, Masson trichrome, and PAS–Alcian Blue reaction.

### Radiological examination of patients with COVID-19

CT images from the COVID-19 and control cases were retrospectively reviewed by a radiologist with more than 10 years of practical experience with chest CT. Interstitial lesions were considered present if there were interstitial reticular opacities with cystic lesions (suggestive of traction bronchiectasis or honeycombing) in the bilateral lower lobes of the lung.

### Statistical analysis

Pearson’s chi-square test or Fisher’s exact test was used to analyze correlations between the interstitial lesions and different parameters. Multivariate analyses were performed to confirm independent correlations and to determine odds ratios by logistic regression analysis. *p* values < 0.05 were considered significant. All analyses were performed using JMP ® Pro 15 (SAS Institute, Cary, NC, USA).

## Results

### Histopathological study

#### Interstitial lung lesion was more frequently detected in the COVID-19 cases

**Fig. 3:**
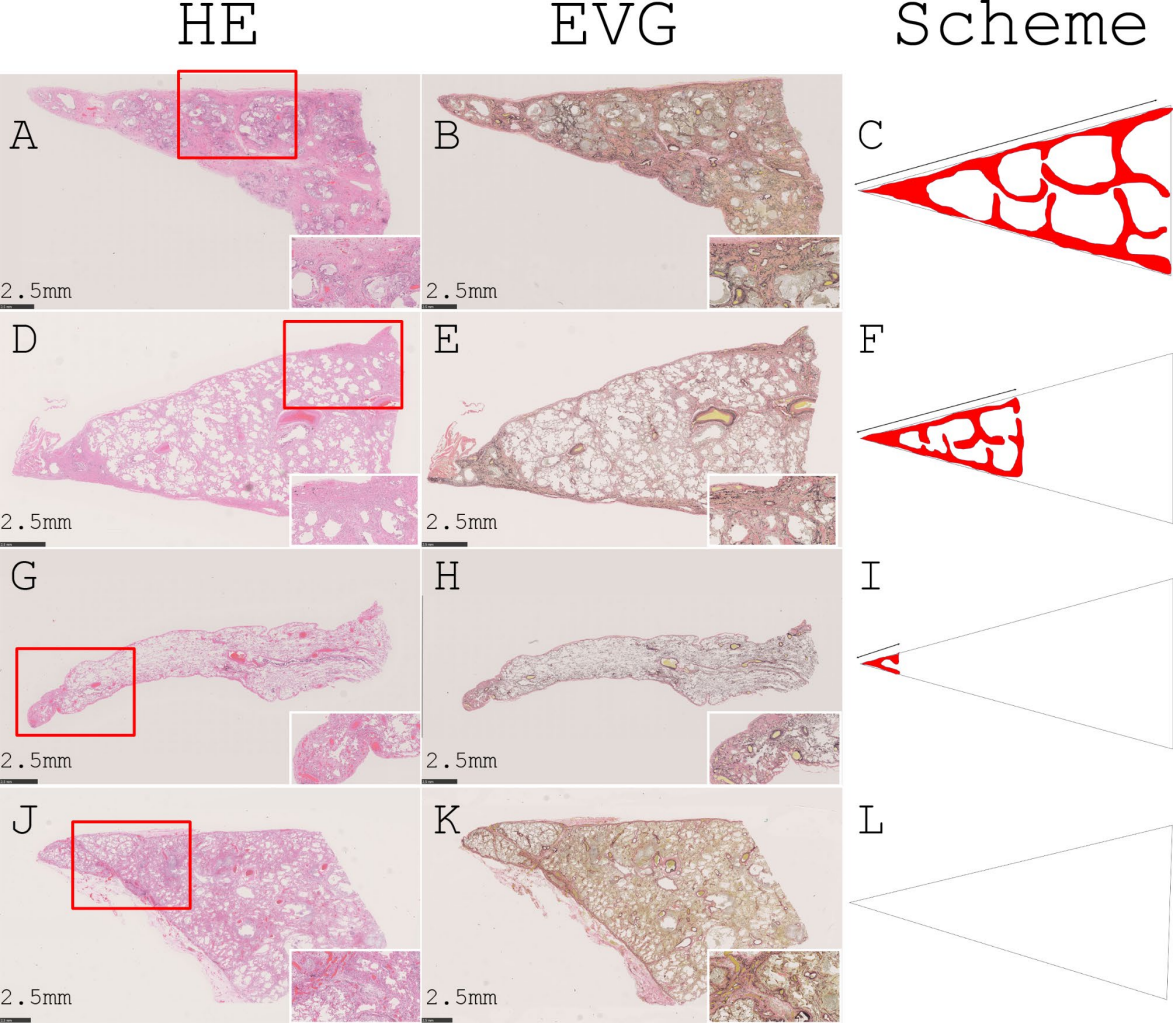
Representative histological photographs and schematic diagrams of different levels of usual interstitial pneumonia (UIP) lesion. A UIP lesion extends throughout the histological section, where normal alveolar structure is lost and dense fibrosis is seen in subpleural and/or perilobular areas (**A, B, C**). In the other case, the UIP lesion is found to extend up to 70% of the area of the section (**D, E, F**). While in the other case, the UIP lesion is only found in small areas (only a few lobules), where dense fibrosis is confined to the tip (**G, H, I**). In the control case, no dense fibrosis suggestive of a UIP lesion is found. However, there is loose fibrosis suggestive of organizing alveolar damage in the alveolar space and on the alveolar septa (**J, K, L**). The inset images are from the red squares in each. We judged the UIP lesions in the top (**A, B, C**) and second (**D, E, F**) panels as “histological UIP”; when a “histological UIP” is seen in the bilateral lungs, we define it as a “subclinical/histological ILD” (**A, D, G, J,** hematoxylin and eosin; **B, E, H, K,** Elastica van Gieson; **C, F, I, L,** scheme)

We focused on the lung bases and carefully examined them for interstitial lesions to detect subclinical ILD since ILD (UIP/IPF) is believed to start from the subpleural area of lung bases [21–24]. As shown in Fig. 3, we found various interstitial lesions with the UIP pattern [25, 26], where alveolar collapse, smooth muscle proliferation and dense fibrosis with occasional fibroblastic proliferation are dominant at the subpleural or peri-lobular area. Interstitial lesion size (expansion level) varied from those that could barely be identified in a microscopic examination to those that could easily be detected in a gross examination (Fig. 3). Thus, we defined “histological UIP” as a lesion with alveolar collapse with fibrosis not confined to the apical region but extending over unequivocally multiple lobules. We diagnosed “subclinical/histological ILD (s/hILD)” if a histological UIP was found in the bilateral lungs in the autopsy cases. “s/hILD” was seen in 13/27 cases (48%) in the COVID-19 group and in 8/65 cases (12%) in the control autopsy group; the difference was statistically significant (*p* = 0.0006, Fisher’s exact test) (Tables 2 and 3). To further confirm the difference, we compared the frequency of an “s/hILD” diagnosis between the COVID-19 and control surgical groups (where cases of only unilateral histological UIP were diagnosed as “s/hILD”). Consistent with the previous results, the frequency was significantly higher in the COVID-19 group (48%, 13/27 versus 10%, 5/48; *p* = 0.0005, Fisher’s exact test) (Tables 4 and 5).

**Table 2:** Comparison of s/hILD frequency and basic characteristics in COVID-19 autopsy cases and in non-COVID-19 autopsy cases, univariate analysis

	COVID-19 autopsy cases (N = 27)	Non-COVID-19 autopsy cases (N = 65)	Univariate analysis
	**% (N)**	**% (N)**	***p*** **-value**
Age			0.7858
Older (≥ 75)	74.07 (20)	75.38 (49)	
Younger (< 75)	25.93 (7)	24.62 (16)	
Sex			1.0000
Male	62.96 (17)	60.00 (39)	
Female	37.04 (10)	40.00 (26)	
Smoking history			1.0000
Severe	57.14 (8)	52.83 (28)	
Light / Never	42.86 (6)	47.17 (25)	
Interstitial lesions			
s/hILD			0.0006*
+	48.15 (13)	12.31 (8)	
−	51.85 (14)	87.69 (57)	
Other interstitial lesion			0.3786
+	11.11 (3)	20.00 (13)	
−	88.89 (24)	80.00 (52)	
Emphysema			0.3598
+	22.22 (6)	13.85 (9)	
−	77.78 (21)	86.15 (56)	

s/hILD: subclinical/histological ILD

*Significant value, *p-*value was calculated by Fisher’s exact test with a public software JMP ver. 15

In univariate analysis, only s/h ILD exhibited a significantly higher frequency in COVID-19 autopsy cases than in non-COVID-19 autopsy cases (*p*-value 0.0006).

**Table 3:** Comparison of s/hILD frequency and basic characteristics in COVID-19 autopsy cases and in non-COVID-19 autopsy cases, multivariate analysis

	Multivariate analysis		
	**Odds ratio**	**95% CI**	***p*** **-value**
Age	0.81	0.25–2.58	0.7203
Sex	0.75	0.26–2.21	0.6055
Interstitial lesions			
s/hILD	7.36	2.36–22.94	0.0006*
Other interstitial lesions	0.54	0.12–2.52	0.4357
Emphysema	2.71	0.70-10.45	0.1475

s/hILD: subclinical/histological ILD

*Significant value, *p-*value was calculated by logistic regression analysis with a public software JMP ver. 15

In multivariate analysis as well, significant results were obtained only for s/h ILD in COVID-19 autopsy cases (*p*-value 0.0006).

**Table 4:** Comparison of s/hILD frequency and basic characteristics in COVID-19 autopsy cases and in non-COVID-19 surgical cases, univariate analysis

	COVID-19 autopsy cases (N = 27)	Non-COVID-19 surgical cases (N = 48)	Univariate analysis
	**% (N)**	**% (N)**	***p*** **-value**
Age			0.0285*
Older (≥ 75)	74.07 (20)	45.83 (22)	
Younger (< 75)	25.93 (7)	54.17 (26)	
Sex			0.8033
Male	62.96 (17)	66.67 (32)	
Female	37.04 (10)	33.33 (16)	
Smoking history			0.7548
Severe	57.14 (8)	64.58 (31)	
Light / Never	42.86 (6)	35.42 (17)	
Interstitial lesions			
s/hILD			0.0005*
+	48.15 (13)	10.42 (5)	
−	51.85 (14)	89.58 (43)	
Other interstitial lesion			0.6972
+	11.11 (3)	8.33 (4)	
−	88.89 (24)	91.67 (44)	
Emphysema			1.0000
+	22.22 (6)	20.83 (10)	
−	77.78 (21)	79.17 (38)	

s/hILD: subclinical/histological ILD

*Significant value, *p-*value was calculated by Fisher’s exact test with a public software JMP ver. 15

In univariate analysis, age and s/h ILD were found to be significantly more frequent in COVID-19 autopsy cases than in non-COVID-19 surgical cases (*p*-value 0.0285, 0.0005).

**Table 5:** Comparison of s/hILD frequency and basic characteristics in COVID-19 autopsy cases and in non-COVID-19 surgical cases, multivariate analysis

	Multivariate analysis		
	**Odds ratio**	**95% CI**	***p*** **-value**
Age	3.14	0.98–10.1	0.0543
Sex	0.71	0.19–2.62	0.6042
Interstitial lesions			
s/hILD	8.88	2.37–33.24	0.0012*
Other interstitial lesions	0.69	0.11–4.45	0.6960
Emphysema	1.33	0.31–5.76	0.7060

s/hILD: subclinical/histological ILD

*Significant value, *p-*value was calculated by logistic regression analysis with a public software JMP ver. 15

In multivariate analysis, significant results were obtained only for s/h ILD in COVID-19 autopsy cases (*p*-value 0.0012).

It was unclear whether the histological UIP lesions developed before or after COVID-19 disease in the COVID-19 group. A previous study on surgical lung biopsies showed that UIP-like lesions appeared after SARS-CoV-2 infection [27]. However, the lesions showed dense hyalinizing fibrosis that appeared much older than the organizing lesion related to COVID-19 pneumonia and was considered to have developed before the onset of COVID-19 pneumonia because patients died soon after the development of the lesions (S1 Table).

#### Interstitial lung lesions in the COVID-19 group could be detected in CT images

**Fig. 4:**
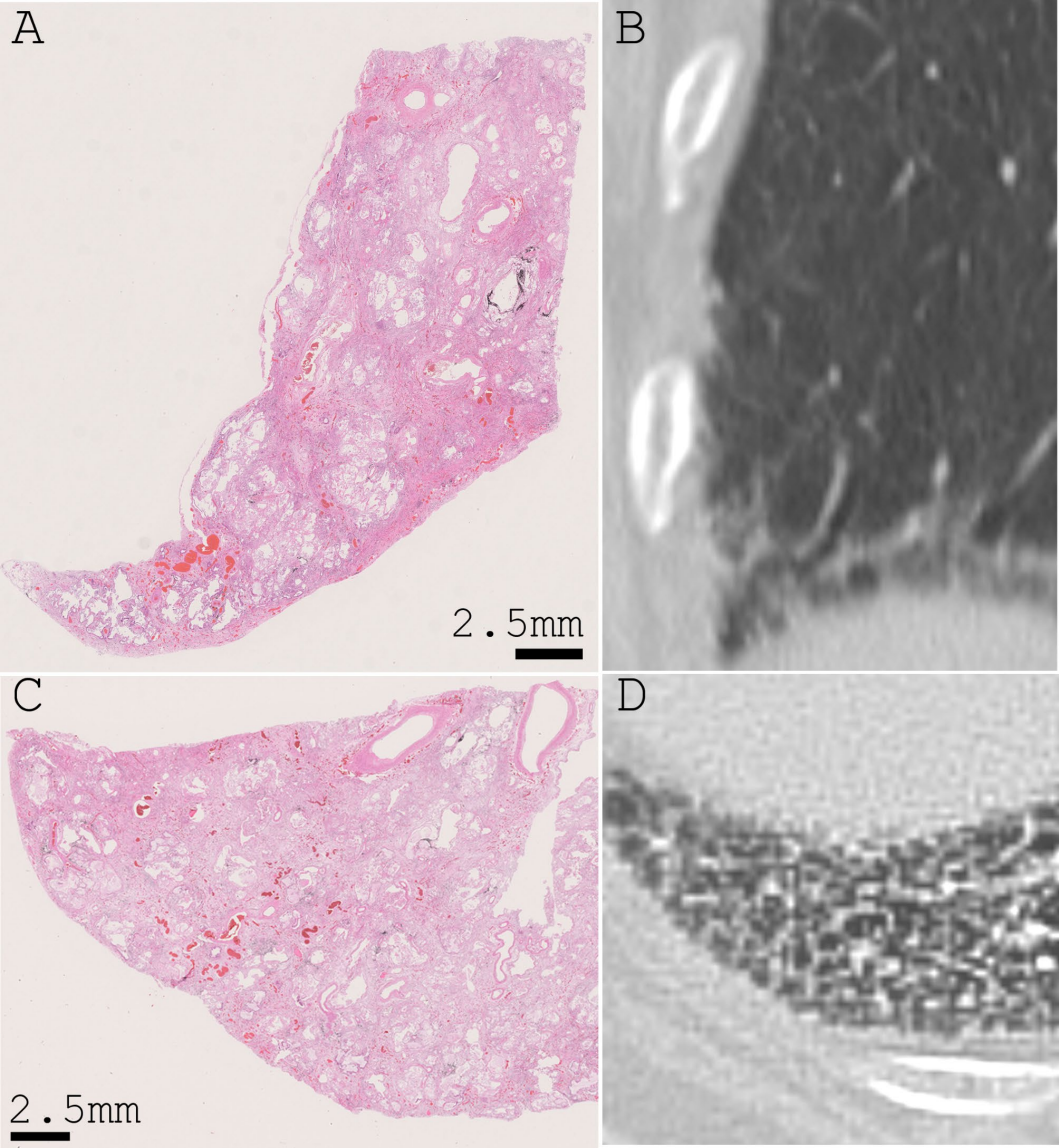
Comparison of subclinical ILD and CT images. Comparative photographs of subclinical ILD in a histological section (**A** and **C**) and corresponding CT (**B** and **D**) from a representative COVID-19 autopsy case (coronal section A and **B**; axial section **C** and **D**). In the left panels (**A** and **C**), a usual interstitial pneumonia lesion is seen as alveolar collapse with dense fibrosis at the subpleural and/or perilobular areas throughout the section. In the right panels (**C** and **D**), a lesion with reticular opacities and small cystic lesions is seen

We reviewed CT images from the COVID-19 cases at multiple time points, including times before COVID-19 onset. Of the series, 15 cases were available for examination for interstitial lesions. Of the remaining 12 unavailable cases, 7 cases could not be evaluated due to extensive, severe organizing DAD that can mask interstitial lesions. Further, one case did not have CT images, and four cases did not have available CT images because the test was conducted in distant hospitals. Interstitial lesions were considered “positive” if there were obvious reticular opacities with occasional cystic lesions (1 cm or larger) in the bilateral lung bases. We defined these radiological findings as “subclinical/radiological ILD (s/rILD)”. Essential information and results from CT examination are summarized in Table 6. “s/rILD” was detected in all cases that had “s/hILD” features (Table 6); the difference was statistically significant (Table 7). The observation suggested that subclinical ILD could be detected in CT images. A representative CT image and the corresponding histological image are shown in Fig. 4.

**Table 6:** Number of times CT was taken before and after the disease and presence of s/rILD and s/hILD in COVID-19 autopsy cases

	Number of times CT was taken			
Case No.	**Before the disease**	**After the disease**	**s/rILD**	**s/hILD**
1	1	0	−	−
2	2	2	−	−
3	2	1	−	−
4	2	1	−	−
5	2	0	+	+
6	2	0	+	−
7	4	1	+	+
8	0	1	−	−
9	0	1	+	−
10	0	4	+	+
11	0	1	+	+
12	0	5	+	+
13	1	0	−	−
14	0	1	−	−
15	0	1	−	−

s/rILD: subclinical/radiological ILD

s/hILD: subclinical/histological ILD

In 15 cases, evaluable CT images with interstitial shadows were present, 8 of which could be compared with pre-COVID CT images

**Table 7:** Agreement between interstitial lung shadows on CT images in COVID-19 autopsy cases

	s/hILD (+) (N = 5)	s/hILD (-) (N = 10)	Univariate analysis
	**% (N)**	**% (N)**	***p*** **-value**
s/rILD			0.0070*
+	100.00 (5)	20.00 (2)	
−	0 (0)	80.00 (8)	

s/rILD: subclinical/radiological ILD

s/hILD: subclinical/histological ILD

*Significant value, *p-*value was calculated by Fisher’s exact test with a public software JMP ver. 15

In all cases with s/hILD, s/rILD are detectable (*p*-value: 0.0070).

### Radiological study

#### Interstitial lung lesion (subclinical/radiological ILD) was related to COVID-19 severity

Finally, to further verify whether “s/rILD” could be a determinant of COVID-19 pneumonia severity, we reviewed CT images from a series of COVID-19 cases in the Yokohama, Japan area for a COVID-19 treatment consortium. We analyzed the relationship between interstitial lesion positivity and COVID-19 pneumonia severity. Severity was evaluated according to the Ministry of Health, Labor, and Welfare (Japan) classification system for COVID-19 severity [28] (S4 Table). “s/rILD” was more frequently found in the group with the moderate II/severe disease than in the moderate I/mild disease (severity was evaluated according to the COVID-19 severity classification system of the Ministry of Health, Labor, and Welfare [Japan]) (moderate II/sever versus moderate I/mild; Fisher exact test, *p* = 0.0333). (S5 Table). These results supported our notion that “s/rILD” could aggravate COVID-19 pneumonia.

## Discussion

A novel and the most notable finding of this study is that a considerable proportion of the patients with severe COVID-19 pneumonia had subclinical interstitial lung lesions, and the frequency was significantly higher than that in the control cases. This finding strongly supports our hypothesis that severe COVID-19 pneumonia could be an exacerbation of subclinical ILD triggered by SARS-CoV-2 infection.

A previous study indicated that small interstitial lung lesions with the UIP pattern (suggestive of early IPF) were often detected in the lung bases of patients with idiopathic acute interstitial pneumonia, and the authors suggested that idiopathic acute interstitial pneumonia could be only an exacerbation of preexisting subclinical ILDs (IPF) [21–24]. Additionally, ILDs, particularly those with the UIP pattern (IPF), have been associated with exacerbation, even if they are only early and focal diseases [22, 24, 29]. In that case, infectious diseases are a common trigger, and viruses such as influenza, human herpes, and cytomegalovirus have been reported to be involved [30–33]. Further, regarding pathogenetic mechanisms, there is an association between severe COVID-19 pneumonia and ILD exacerbation. Initially, COVID-19 pneumonia was considered to be mainly caused by direct cellular damage of type 2 pneumocytes by SARS-CoV-2 infection [34, 35]. However, immunohistochemical studies have shown that virus-infected cells were detected only focally, although DAD affected the entire lungs [36, 37]. Therefore, there should be another mechanism, such as excessive immunological responses. Notably, elevations in the levels of several cytokines have been reported [38–42], and it is currently widely accepted that some cytokines (e.g.; IL-6, IL-8 and others) is related to the development of severe COVID-19 pneumonia [38–42]. Moreover, it is considered that the same cytokines (IL-6 and IL-8) are also essential to trigger ILD exacerbation [43–45]. Thus, these observations seem to support our hypothesis that SARS-CoV-2 could trigger the exacerbation of hidden ILD and the development of DAD through the hyperactivation of cytokines, that is, severe COVID-19 pneumonia.

In addition to the pathological examination, we performed radiological examination using CT images from another series of patients with COVID-19. The results of that analysis—patients with interstitial lesions on CT images had more severe disease than those who did not—further support our hypothesis. Recently, a radiological term “interstitial lung abnormalities (ILA)” was proposed to describe subclinical hidden ILDs on CT images [46, 47]. ILA is defined as an interstitial abnormality detected in patients without a clinical history of ILDs. ILA is seen in at least 5% of the field in any slice of a whole lung CT image [46]. Studies have reported that some cases (up to 70%) of ILA progressed to clinical disease (i.e., equivalent to overt ILDs) [48–51]. Studies on the radiological-pathological associations in interstitial lung lesions suggested that a considerable fraction of ILAs could include pathological UIP lesions [49, 52]. We defined “s/rILD” as an area (≥ 10 mm) of reticular opacities (occasionally with cystic changes = traction bronchial ectasia) in the bilateral lung bases. Our definition of “s/rILD” may be the same as that of “ILA” in a broad sense. In any case, subclinical ILD (conceptually equivalent to ILA) may be a risk factor for severe COVID-19 pneumonia. This is a novel finding of the present study, and we believe it is important to understand the potential pathological bases of DAD in various situations.

### Limitations

The limitations of this study are (1) we could not examine a large number of autopsy cases because autopsies have been restricted for infection control purposes; (2) in the autopsy cases, it was difficult to distinguish among preexisting hidden interstitial lesions, drug-induced fibrotic lesions, and COVID-19-related scarring lesions, particularly in those with long disease periods (more than two months; only one such cases was present out of the 27 cases). In this study, we explored whether the presence or absence of s/hILD varied based on the time between exacerbation and death, as well as the usage of specific medications (anti-viral drugs, steroids, immunosuppressive drugs, chloroquine, and antibiotics). Our analysis revealed no significant differences in relation to the duration from exacerbation to death (N = 27, logistic regression analysis, p = 0.0913) or the use of anti-viral drugs (chi-square test, p-value undefined), steroids (chi-square test, p = 0.4815), immunosuppressive drugs (chi-square test, p = 0.1283), or antibiotics (chi-square test, p = 1.0000); (3) we could not histologically examine lung tissues from patients who survived from COVID-19 without severe pneumonia as controls. It is impossible to examine autopsied lungs from patients who survived COVID-19. Instead, we used autopsy and surgical cases unrelated to COVID-19 as a control group in this study. Additionally, we examined interstitial lung abnormalities that may be equivalent to histological UIP in CT images from COVID-19-survivors and analyzed their relationship with disease severity; (4) in the radiological study, hidden interstitial lesions may not have been detectable in the cases with extensive DAD and not all cases could be evaluated by high-resolution computed tomography. We understand the influence of these limitations on the results.

## Conclusion

This is the first study to investigate potential risk factors for severe COVID-19 pneumonia through pathological examinations of autopsy specimens. Our results indicate that subclinical ILDs could be an important risk factor for severe COVID-19 pneumonia. We believe our observations can assist the development of a risk assessment system for fatal COVID-19 pneumonia by using high-resolution CT images to detect ILA.

### Electronic supplementary material

Below is the link to the electronic supplementary material.

Supplementary Material 1

Supplementary Material 2

Supplementary Material 3

Supplementary Material 4

Supplementary Material 5

Supplementary Material 6

## Data Availability

The datasets used and analyzed during this study are available from the corresponding author on reasonable request.

## Abbreviations

COVID-19: Coronavirus disease 2019
ILD: Interstitial lung disease
IPF: Interstitial pulmonary fibrosis
UIP: Usual interstitial pneumonia
DAD: Diffuse alveolar damage
CT: Computed tomography
s/hILD: Subclinical/histological ILD
s/rILD: Subclinical/radiological ILD
ILA: Interstitial lung abnormalities
