# Supplementary material for: Frequency of subclinical interstitial lung disease in COVID-19 autopsy cases: potential risk factors of severe pneumonia
Source: BMC Pulm Med. 2023 Oct 27;23:408. doi: 10.1186/s12890-023-02692-1 (PMC10612296; doi:10.1186/s12890-023-02692-1)
Supplement: Supplementary file 6 — Supplementary Material 6 [file 12890_2023_2692_MOESM6_ESM.docx]

**Additional files**

**S1 Table**

**S1_Table_revised.xlsx**

**Essential information of autopsy cases (COVID-19) examined in the present study**

This table summarizes the essential baseline information of the COVID-19 autopsy cases.

**S2 Table**

**S2_Table.xlsx**

**Essential information of autopsy cases (non-COVID-19) examined in the present study**

This table summarizes the essential information of the control autopsy cases.

**S3 Table**

**S3_Table.xlsx**

**Essential information of surgical cases (non-COVID-19) examined in the present study**

This table summarizes the essential information of the control surgical cases.

**S4 Table**

**S4_Table.xlsx**

**The Ministry of Health, Labour, and Welfare (Japan) classification system for COVID-19 severity**

This table summarizes the classification of COVID-19 severity used in Japan.

**S5 Table**

**S5_Table.xlsx**

**Comparison of COVID-19 severity status and presence of s/rILD**

This table shows the association between COVID-19 severity and s/rILD in the 260 COVID-19 patients in the Kanagawa Cardiovascular and Respiratory Center.
